# The immune phenotype of tongue squamous cell carcinoma predicts early relapse and poor prognosis

**DOI:** 10.1002/cam4.3440

**Published:** 2020-10-13

**Authors:** Giuseppe Troiano, Corrado Rubini, Lucrezia Togni, Vito Carlo Alberto Caponio, Khrystyna Zhurakivska, Andrea Santarelli, Nicola Cirillo, Lorenzo Lo Muzio, Marco Mascitti

**Affiliations:** ^1^ Department of Clinical and Experimental Medicine University of Foggia Foggia Italy; ^2^ Department of Biomedical Sciences and Public Health Marche Polytechnic University Ancona Italy; ^3^ Department of Clinical Specialistic and Dental Sciences Marche Polytechnic University Ancona Italy; ^4^ Dentistry Clinic National Institute of Health and Science of Aging INRCA Ancona Italy; ^5^ Melbourne Dental School The University of Melbourne Melbourne Vic. Australia

**Keywords:** head and neck, immunity, oral squamous cell carcinoma, prognosis, tongue squamous cell carcinoma

## Abstract

**Background:**

In patients with squamous cell carcinoma of the oral tongue (OTSCC), current tumor node metastasis staging system fails to identify at‐risk patients associated with early relapse and poor prognosis despite complete surgical resection. Given the importance of tumor‐infiltrating lymphocytes (TILs) in the development of cancers, here we investigated the prognostic significance of the immune phenotype in OTSCC.

**Methods:**

Hematoxylin‐eosin stained sections of OTSCCs from 211 patients were evaluated. Cancers were classified as (a) immune‐inflamed when TILs were found next to tumor cell nests; (b) immune‐excluded when TILs were found in the stroma, outside the tumor; and (c) immune‐desert for tumors lacking lymphocyte infiltrate. The prognostic significance of these immune phenotypes classes was investigated. Data were further validated on an independent cohort from The Cancer Genome Atlas database.

**Results:**

Immune‐desert phenotype was the least represented group of OTSCCs in our cohort (11.8%) and served as an independent prognostic factor. Patients with immune‐desert tumors exhibited worse disease‐specific survival (HR = 2.673; [CI: 95% 1.497‐4.773]; *P* = .001), overall survival (HR = 2.591; [CI: 95% 1.468‐4.572]; *P* = .001), and disease‐free survival (HR = 2.313; [CI: 95% 1.118‐4.786]; *P* = .024) at multivariate analysis.

**Conclusions:**

We identified a specific subgroup of OTSCCs with poor prognosis. Tumor‐infiltrating lymphocytes density and localization could serve as an integrative parameter to the current staging system and inform the selection of most appropriate treatments. In particular, the tumor immune phenotype could improve the stratification of patients with more aggressive disease.

## Graphical abstract

In patients with squamous cell carcinoma of the oral tongue (OTSCC), current tumor node metastasis staging system fails to identify at‐risk patients associated with early relapse and poor prognosis despite complete surgical resection. Given the importance of tumor‐infiltrating lymphocytes in the development of cancers, here we investigated the prognostic significance of the immune phenotype in OTSCC.

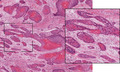

## 1 INTRODUCTION

Oral squamous cell carcinoma (OSCC) represents one of the most common malignancies in humans with almost 400 000 new cases diagnosed annually worldwide.^1^ In certain geographical regions, such as areas of the Indian subcontinent and South East Asia, oral cancer is the most common neoplasm.^2^ Oral squamous cell carcinoma encompasses cancers developing in various sites of the oral cavity, and intraoral tongue is the most commonly involved subsite, accounting for about 40% of all OSCC cases.^3^ Interestingly, recent studies have shown that oral tongue squamous cells carcinoma (OTSCC) exhibits peculiar molecular and clinical behavior compared to OSCC from other subsites of the oral cavity.^4^, ^5^, ^6^, ^7^ If not properly recognized, the presence of this potential “anatomical bias” may hinder the interpretation and clinical translation of OSCC data.^8^, ^9^

The surgical resection of primary OTSCC, with or without neck dissection, is still considered the gold standard of treatment.^10^ Other treatment approaches include radiation therapy and/or systemic therapy (chemotherapy or targeted therapy).^11^ Both medical and surgical treatment protocols are dictated by the assessment of disease staging, which reflects an estimate of the biological and clinical behavior of the tumor. Unfortunately, OTSCC is characterized by a high rate of local and regional recurrence after primary treatment, which strongly decreases patients' survival rates.^12^, ^13^ No prognostic biomarkers have been validated so far to stratify patients on the basis of OTSCC‐related risk of recurrence and death. The main predictor of clinical outcome is still represented by tumor size, lymph node involvement, and presence of metastasis (tumor node metastasis staging), which is used to inform prognostic estimates and treatment planning.^14^ The 8th edition of the AJCC/UICC staging system released in 2017 included main changes such as the adoption of the depth of invasion (DOI) and extranodal extension (ENE) as parameters for staging determination.^15^, ^16^ Despite such new system outperform the 7th AJCC staging edition in the prognostic ability,^17^, ^18^, ^19^ accumulating evidences show that the predictive capability of 8th AJCC staging system still need to be improved in order to obtain a more robust prognostic stratification.^20^ In particular, the 8th AJCC staging system fails to identify OTSCC patients characterized by early relapse and poor prognosis despite complete surgical resection and no evidence of residual tumor burden.^21^ Therefore, it is imperative to find new prognostic biomarkers that can inform appropriate clinical decisions based on individual bio‐clinical characteristics, rather than on population averages.^18^, ^22^

One of the well‐established hallmarks of cancer is the ability of cancer cells to evade the host immune system.^23^, ^24^ Several studies conducted in recent years suggest that tumor‐infiltrating lymphocytes (TILs) and their spatial organization in the tumor microenvironment play a crucial role in cancer progression.^25^ In particular, solid tumors have been histologically classified in three immune profiles according to the distribution of T cells, also known as tumor‐immune phenotype: immune‐inflamed, immune‐excluded, and immune‐desert phenotypes.^26^ Immune‐inflamed tumors are characterized by the presence of a dense immune cell infiltrate. In this profile, immune cells are next to the tumor cells. The immuno‐excluded profile is also characterized by an abundant presence of immune cells which, unlike inflamed tumors, do not penetrate the parenchyma of the neoplasm and are retained in the stroma. Lastly, the immune‐desert phenotype is characterized by an almost total absence of immune cells in either the parenchyma or the stroma of the tumor.^27^ Although this classification is now widely accepted, few studies have explored the clinical impact of the immune profile in different cancer types.^28^ Hence, the aim of this study was to investigate the prognostic role of the immune phenotype in OTSCC samples.

## 2 MATERIALS AND METHODS

### 2.1 Case selection

The present retrospective study considered tumor samples obtained from a cohort of randomly selected patients with OTSCC who were treated with surgical resection with curative intent at the at the Department of Maxillofacial Surgery, “Ospedali Riuniti” General Hospital (Ancona, Italy), between 1997 and 2014. The clinical and pathological data were retrieved from the archives of the Sections of Pathology, Marche Polytechnic University, Italy, by a single operator (LT).

Inclusion criteria were: (a) primary OTSCC (International Classification of Disease‐10 diagnosis codes: C02.0, C02.1, C02.2, and C02.3); (b) age above 18 years; (c) no human papilloma virus (HPV) infection (assessed using HPV 16‐specific fluorescence in situ hybridization and p16^Ink4a^‐specific immunohistochemistry); (d) follow‐up data of at least 3 years for alive patients.

Exclusion criteria were: (a) neoadjuvant therapy (ie, preoperative chemotherapy or preoperative radiation therapy); (b) OTSCC cases involving other anatomical sites and tumors of uncertain origin; (c) relapsed or secondary primary OTSCC; (d) OTSCC patients with immediate postoperative death. Randomly selected OTSCC patients who fulfilled the inclusion and exclusion criteria were further stratified by pathological stage in order to assign the same number of patients in each pathological stage group (pTNM). The staging classification was revised by two expert pathologists (CR and MM) blinded to clinical data, according to the 7th and 8th editions of the AJCC Cancer Staging Manual^29^, ^30^ and the 4th edition of the World Health Organization classification of Head and Neck tumors.^31^ Clinical examined endpoints were disease‐specific survival (DSS), overall survival (OS), and disease‐free survival (DFS). Follow‐up time was calculated from the time of surgical operation to the time of death for cancer or last follow‐up visit for DSS, while for OS also death for other causes was taken into consideration. For DFS, follow‐up time was calculated from the date of surgical operation to the date of recurrence or the date of the last visit without recurrence.

Informed consent was obtained from all included patients, and the study was conducted in accordance with the “Ethical Principles for Medical Research Involving Human Subjects” statement of the Helsinki Declaration.^32^ This study received ethical approval from the institutional review board of Marche Polytechnic University, Italy (CERM 2019‐308). The study was conducted according to the REMARK checklist.^33^

### 2.2 Histopathologic evaluation

Routine 4‐μm hematoxylin‐eosin (H&E) stained sections, obtained from formalin‐fixed, paraffin‐embedded blocks of the primary tumor specimens, were carried out from the most invasive part of the primary tumor (ie, the same slides routinely used to assess the T status). The density and localization of lymphocytes were determined, based on the recommendation of the International TILs Working Group.^34^

Briefly, for each patient, one H&E stained section was considered. A full assessment of the tumor area was initially conducted by light microscopy at low magnification. Subsequently, five high‐power fields with a magnification of ×200 (ocular ×10, with an objective of ×20) were randomly selected. The presence of TILs was evaluated in the stromal compartment within the borders of invasive tumor, by considering all the mononuclear cells. In particular, the percentage of TILs was estimated based on the percentage of the area occupied by mononuclear cells over the stromal area both around the tumor border and inside the tumor mass in proximity to the tumor cells. Necrotic areas have been left out of the microscopic scoring field whenever possible, if this was not possible, these areas have been ignored for scoring. The area percentage was estimated per 10‐fold, from 0% to 100%, per image‐field. For each patient, the mean value of scored percentages was considered.

**FIGURE 1:**
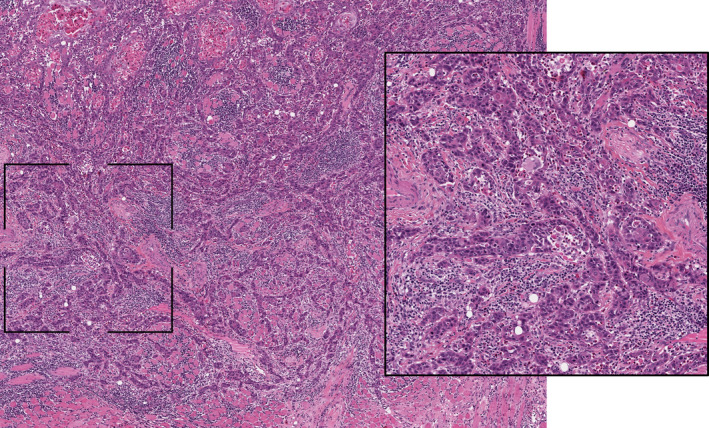
Representative pictures of immune‐inflamed phenotype in oral tongue squamous cells carcinoma (hematoxylin‐eosin staining, ×20 magnification). The inset area of greater magnification showed the presence of a dense T cell infiltrate both in the tumor mass and in the stromal area

**FIGURE 2:**
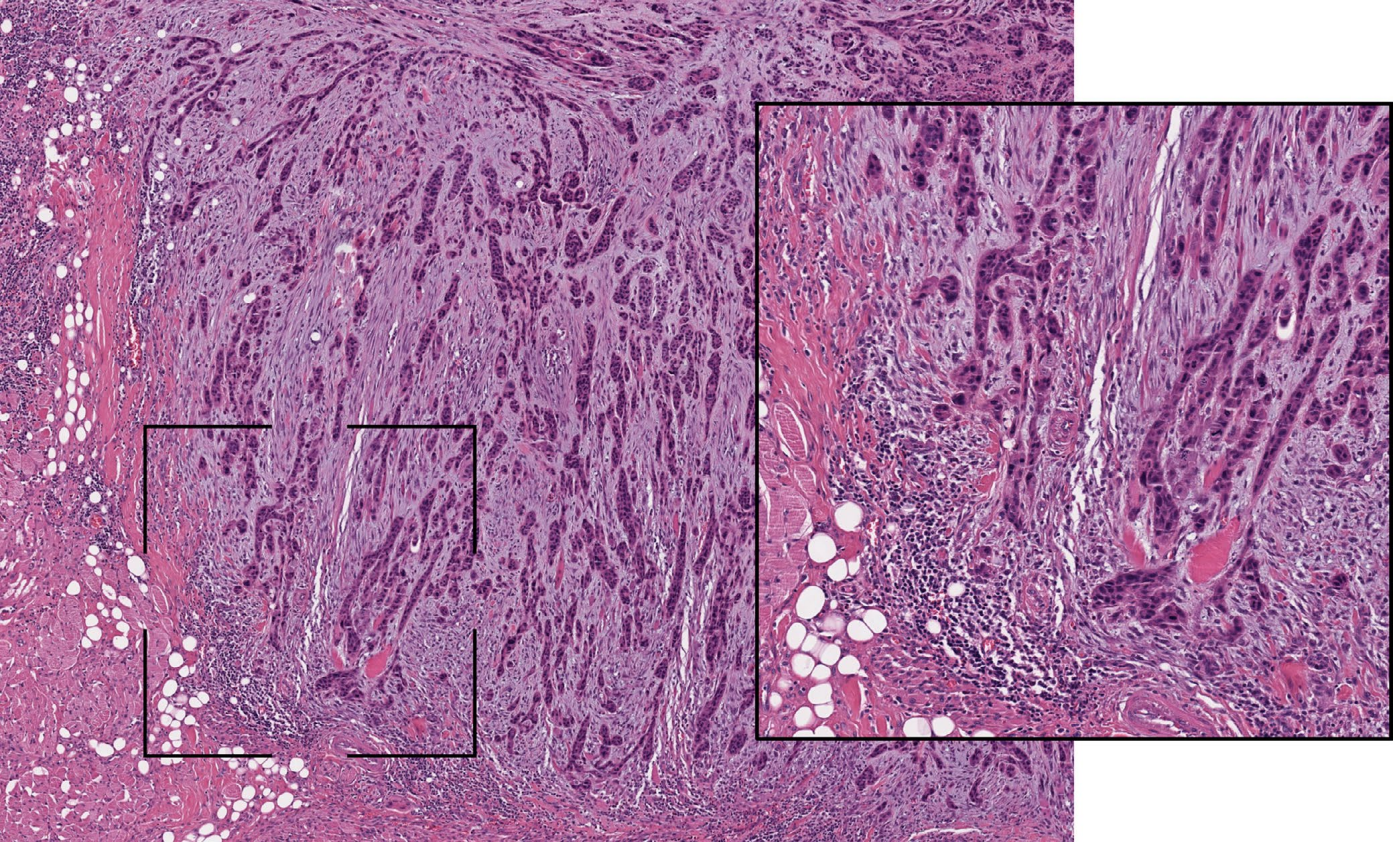
Representative pictures of immune‐excluded phenotype in oral tongue squamous cells carcinoma (hematoxylin‐eosin staining, ×20 magnification). The inset area of greater magnification showed the presence of a T cell infiltrate only in the stromal area around the tumor border

**FIGURE 3:**
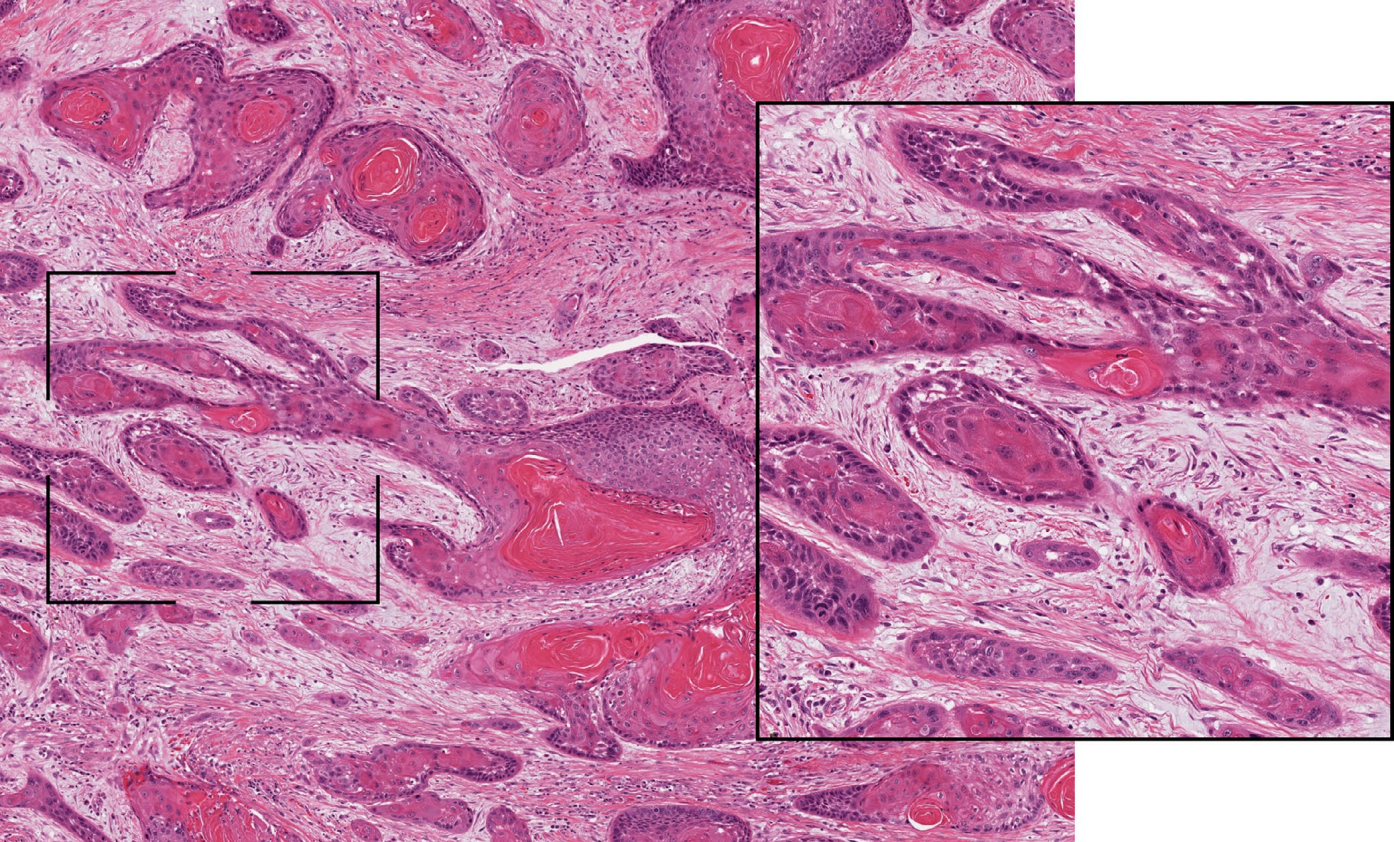
Representative pictures of immune‐desert phenotype in oral tongue squamous cells carcinoma (hematoxylin‐eosin staining, ×20 magnification). The inset area of greater magnification showed the presence of a negligible number of lymphocytes both in the tumor mass and in the stromal area

Based on the results, patients were divided into the following groups: (a) Group 1 (“immune‐inflamed phenotype” group) if the mean percentage of lymphocytes detected inside the tumor mass in proximity to the tumor cells was ≥10%, regardless the presence of lymphocytes in the stromal area around the tumor border (Figure 1); (b) Group 2 (“immune‐excluded” group) if the mean percentage of lymphocytes detected in the stromal area around the tumor border was ≥10%, but there was a negligible amount of lymphocytes inside the tumor mass (<10%) (Figure 2); and (c) Group 3 (“immune‐desert phenotype” group) if the mean percentage of lymphocytes detected both in the tumor mass and in the stromal area was negligible (<10%) (Figure 3). Histological analysis of immune‐phenotype was performed by two authors (CR and MM) in an independent, each author gave a judgment on the belonging of each specimen to a single class of immune‐phenotype. Such independent scores were used to assess the degree of agreement between the observers by calculating a Cohen's Kappa. Subsequently a joint session with a third author (GT) was scheduled in order to give a final judgment on the allocation of the specimen to a single class of immune‐phenotype in cases of disagreement between the two pathologists; such final “score” was then used to perform all the other statistical analysis. Each specimen was analyzed three times. In order to perform an external analysis about the predictive capability of the immune‐phenotype in OTSCC, the Cancer Slide Digital Archie (CDSA) was accessed and analyzed. CDSA is a web‐based platform, which collects digital pathological data from The Cancer Genome Atlas (TCGA) database. Codes were input on https://cancer.digitalslidearchive.org/ and pathological slides were analyzed and classified according to the abovementioned parameters. Clinic‐pathological data were downloaded from Genomic Data Commons Data Portal (https://portal.gdc.cancer.gov/), and patients who underwent neoadjuvant therapy were excluded from the analysis.

### 2.3 Statistical analysis

All the statistical analyses were performed using SPSS statistical software 21.0. Primary endpoint was to detect any significant difference in the survival rate between “immune‐desert phenotype” patients (Group 3) and “non‐immune‐desert phenotype” patients (Group 1 + 2). For this reason, sample size was calculated to evaluate DSS, OS, and DFS with a hazard ratio of 2 (two‐sided 5% significance level for the log‐rank test and a power of 80%). A ratio of 1 to 4 (Group 3 vs Group 1 + 2) was used to estimate a priori the proportion of subjects with immune‐desert phenotype, considering a 10% dropout rate.^35^ Sample size estimation showed that the number of patients required was 207 patients. Normal distribution of age among the three immune‐phenotype groups was explored through Shapiro‐Wilk normality test and the Kolgomorov‐Smirnoff test. Hence, the nonparametric test of Mann‐Whitney was used in order to investigate the age difference among groups.

Trend difference among immune‐phenotypes and the other categorical variables (stage, gender, grading, and perineural invasion) were explored using cross‐classification tables and Chi‐Squared test. Kaplan‐Meier method was used to estimate the OS rates, comparing results by means of the log‐rank test and built survival curves. In addition, univariate and multivariate Cox regression hazard models were built in order to assess the association among predictive variables (immunophenotypes, stage, grade, perineural invasion, and gender) and their influences on the prognostic outcomes (DSS, OS, and DFS), the proportional hazard assumption was checked with the test of nonzero slope in a generalized linear regression of the scaled Schoenfeld residuals on time.

## 3 RESULTS

### 3.1 Demographic and clinicopathological variables

A total of 211 OTSCC patients' samples admitted and treated at the Department of Maxillofacial Surgery, “Ospedali Riuniti” General Hospital, Ancona, Italy, in the period between 1997 and 2014 were included in this study. All these patients had been staged according to the 7th AJCC staging system. In addition, for 139 OTSCC patients' information about DOI and ENE were available, and patients were “restaged” according to the 8th AJCC staging system. In the total cohort of 211 patients, (137/211) 64.9% were males and (74/211) 35.1% were females; the average age of the cohort was 64.15 ± 14.1 years. The histological analysis revealed as most of OTSCC tumors had an immune‐excluded phenotype (109/211, 51.7%), inflamed tumors represented the second most common groups (77/211, 36.5%), while immune‐desert OTSCC were the less represented group (25/211, 11.8%). Details on other clinic‐pathological characteristics of the studied cohort are available in Table 1. No significant correlation was detected between immune‐phenotype and patients' clinic‐pathological variables, such as stage, grading, gender, and perineural invasion (Material S1).

**TABLE 1:** Clinical and pathological characteristics of the patients included in this study (N = 211)

Clinical and pathological data		
Parameters	No.	%
Gender		
Male	137	64.9
Female	74	35.1
Mean age at diagnosis (years ± SD)	64.1 ± 14.1	
Grading		
G1	38	18.4
G2	113	53.6
G3	60	28.4
7th AJCC edition		
Stage I	51	24.2
Stage II	60	28.4
Stage III	48	22.7
Stage IV	52	24.6
8th AJCC edition		
Stage I	28	20.4
Stage II	31	22.6
Stage III	26	19.0
Stage IV	52	38.0
Perineural invasion		
No	83	39.3
Yes	128	60.7
Immune‐phenotype		
Inflamed	77	36.5
Excluded	109	51.7
Desert	25	11.8

Abbreviations: AJCC, Americant Joint Committee on Cancer; no., number.

### 3.2 Survival analysis of immune‐phenotype in OTSCC patients

**FIGURE 4:**
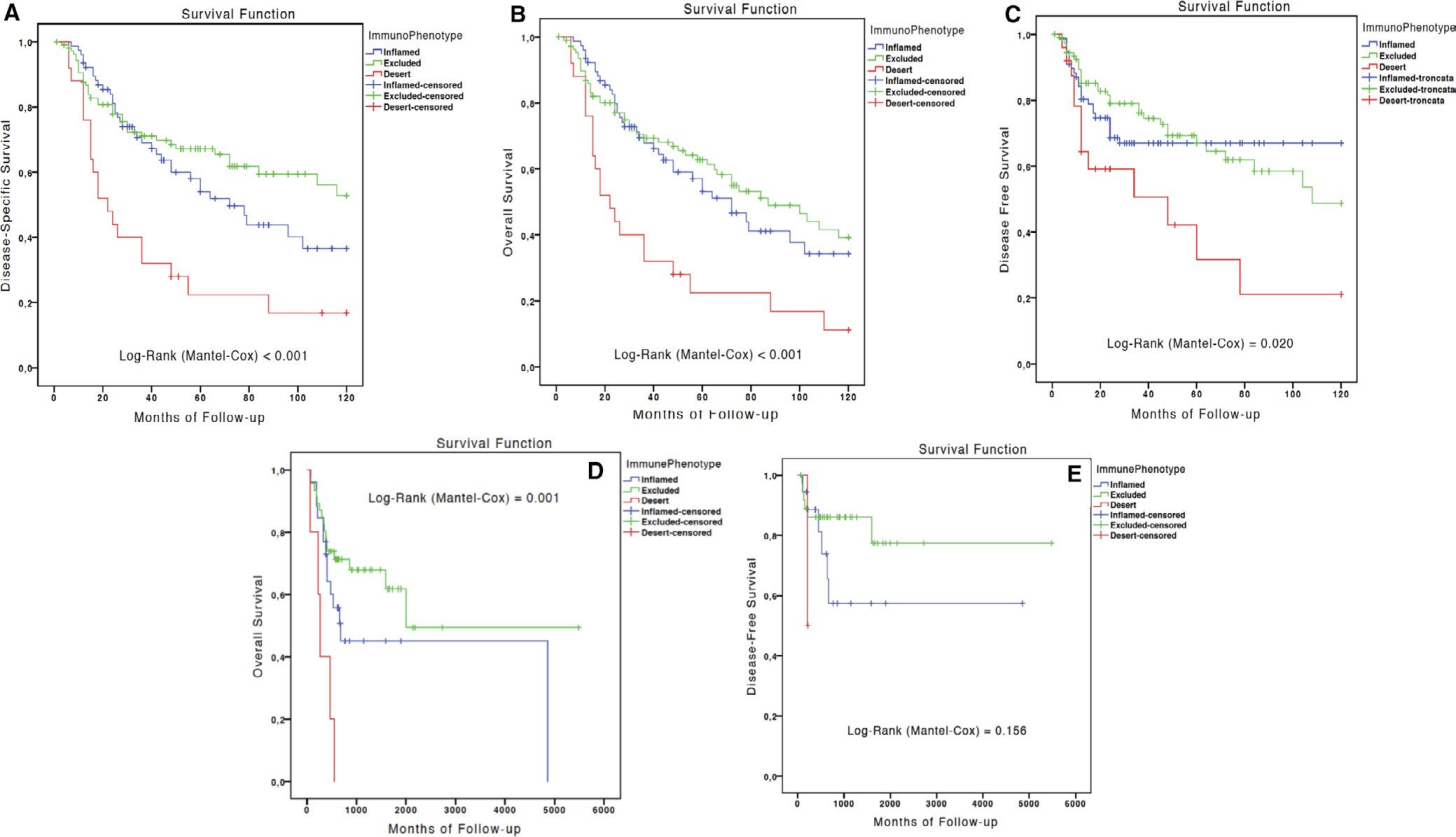
A‐E, Kaplan‐Meier curves for disease‐specific survival (A), overall survival (OS) (B), and disease‐free survival (DFS) (C) in the Italian cohort; and OS (D) and DFS (E) in the The Cancer Genome Atlas cohort

Results of univariate survival analysis performed on the whole cohort of 211 patients showed that OTSCC patients with an immune‐desert phenotype had lower likelihood of survival compared to the other groups: DSS (HR = 2.309; [CI: 95% 1.335‐3.995]; *P* = .003), OS (HR = 2.308; [CI: 95% 1.352‐3.939]; *P* = .002), and DFS (HR = 2.241; [CI: 95% 1.139‐4.411]; *P* = .020). Univariate Kaplan‐Meier curves for DSS, OS, and DFS in our cohort and curves for OS and DFS for TCGA cohort are shown in Figure 4. Other variables that significantly influence OS and DFS at univariate analysis were: 7th AJCC stage, grade, perineural invasion, age, and gender. On the basis of these results, a multivariate Cox proportional hazard model was built including the significant variables (immune‐phenotype, 7th AJCC stage, grade, perineural invasion, and gender). Results of such multivariate analysis confirmed the significant association between immune‐desert phenotype and both DSS (HR = 2.673; [CI: 95% 1.497‐4.773]; *P* = .001) (Table 2) and OS (HR = 2.591; [CI: 95% 1.468‐4.572]; *P* = .001). In addition, applying the same multivariate model, the immune‐desert phenotype resulted to be an independent prognostic factor for DFS (HR = 2.313; [CI: 95% 1.118‐4.786]; *P* = .024) (Table 3). It is worth noting that tumor grade lost its statistical significance in the multivariate model, while 7th AJCC Stage, perineural invasion, and gender all resulted to correlate with a worse DSS.

**TABLE 2:** Results of the univariate and multivariate survival analysis (Cox Proportional Hazard model) for the outcome disease‐specific survival on the Italian cohort (*P < .05)

Variable	Univariate analysis			Multivariate analysis		
	HR	95% CI	*P*‐value	HR	95% CI	*P*‐value
Age	1.013	0.998‐1.029	.080			
Gender						
Female	1			1		
Male	1.756	1.110‐2.780	.016*	1.695	1.026‐2.801	.039*
Grade						
G1	1		.033*	1		.572
G2	0.992	0.543‐1.180	.978	0.702	0.362‐1.360	.294
G3	1.759	0.940‐3.291	.077	0.715	0.339‐1.506	.378
7th AJCC edition						
Stage I	1		<.001*	1		.007*
Stage II	1.995	0.997‐3.992	.051	1.804	0.833‐3.907	.134
Stage III	2.388	1.174‐4.857	.016*	1.397	0.602‐3.239	.436
Stage IV	4.293	2.237‐8.236	<.001*	3.117	1.439‐6.750	.004*
Perineural invasion						
No	1			1		
Yes	2.443	1.506‐3.964	<.001*	2.048	1.172‐3.581	.012*
Immune‐phenotype						
Inflamed	1		<.001*	1		<.001*
Excluded	0.750	0.475‐1.185	.218	0.884	0.540‐1.448	.625
Desert	2.309	1.335‐3.995	.003*	2.673	1.497‐4.773	.001*

Abbreviation: AJCC, Americant Joint Committee on Cancer.

**TABLE 3:** Results of the univariate and multivariate analysis (Cox Proportional Hazard model) for the outcomes Disease‐Free Survival on the Italian cohort (*P < .05)

Variable	Univariate analysis			Multivariate analysis		
	HR	95% CI	*P*‐value	HR	95% CI	*P*‐value
Age	0.998	0.981‐1.015	.788			
Gender						
Female	1			1		
Male	1.159	0.702‐1.913	.565	1.030	0.601‐1.762	.915
Grade						
G1	1		.422	1		.475
G2	1.600	0.750‐3.415	.224	1.668	0.732‐3.799	.223
G3	1.694	0.745‐3.857	.209	1.531	0.598‐3.921	.735
7th AJCC edition						
Stage I	1		.700	1		.964
Stage II	0.778	0.400‐1.513	.459	0.943	0.453‐1.966	.876
Stage III	1.143	0.593‐2.203	.690	1.139	0.541‐2.396	.732
Stage IV	1.085	0.563‐2.091	.807	1.032	0.487‐2.185	.935
Perineural invasion						
No	1			1		
Yes	1.370	0.828‐2.266	.220	1.242	0.694‐2.220	.466
Immune‐phenotype						
Inflamed	1		.026*	1		.028*
Excluded	0.956	0.563‐1.624	.869	0.937	0.537‐1.635	.819
Desert	2.241	1.139‐4.411	.020*	2.313	1.118‐4.786	.024*

Abbreviation: AJCC, Americant Joint Committee on Cancer.

Next, a new model which excluded tumor grade from analysis was built for the subgroup of 139 patients “restaged” according to the 8th AJCC staging system; similarly, results of this analysis revealed a significant worse DSS (HR = 2.280; [CI: 95% 1.107‐4.696]; *P* = .025) and OS (HR = 2.299; [CI: 95% 1.115‐4.742]; *P* = .024) for immune‐desert patients. Regarding DFS, results for the immune‐desert phenotype were close to the threshold of statistical significance (HR = 2.146; [CI: 95% 0.941‐4.893]; *P* = .070), while none of the other clinic‐pathological variables correlated with a worse DFS (Table 4). In order to analyze the results obtained on our internal cohort, digital pathological slides and clinical data from the TCGA database were evaluated from the same researchers (MM and CR) who performed the analysis on authors' cohort. A total of 76 patients' code from Head and neck TCGA database fulfilled the inclusion criteria and were included in the analysis. In this cohort of patients, immune‐desert OTSCCs showed a worse prognosis at univariate analysis: OS (HR = 3.354; [CI: 95% 1.187‐9.479]; *P* = .022). However, due to the limited number of immune‐desert phenotype obtained from the TCGA database, these results were not significant at the multivariate analysis OS (HR = 2.634; [CI: 95% 0.615‐11.272]; *P* = .192). Indeed, all the five immune‐desert patients were classified in Stage 4; therefore, multivariate analysis was unable to determine whether the poor prognosis was due to the advanced stage or the immune‐desert phenotype (Material S2). Taken together, these results demonstrate that the immune‐phenotype, particularly immune‐desert tumors, is an independent prognostic factor in OTSCC patients.

**TABLE 4:** Results from the multivariate analysis for the outcomes Disease‐Specific and Disease‐Free survival including only patients staged according to the 8th AJCC edition (*P < .05)

Variable	Multivariate analysis					
	Disease‐specific survival			Disease‐free survival		
	HR	95% CI	*P*‐value	HR	95% CI	*P*‐value
Gender						
Female	1			1		
Male	1.913	1.001‐3.655	0.050	1.276	0.648‐2.510	0.481
8th AJCC edition						
Stage I	1		0.007*	1		0.614
Stage II	1.699	0.470‐6.140	0.419	0.615	0.228‐1.657	0.336
Stage III	3.013	0.882‐10.289	0.078	1.190	0.472‐3.003	0.713
Stage IV	5.101	1.712‐15.205	0.003*	0.851	0.363‐1.996	0.711
Perineural invasion						
No	1			1		
Yes	1.871	0.982‐3.565	0.057	1.341	0.672‐2.674	0.405
Immune‐phenotype						
Inflamed	1		0.043*	1		0.026*
Excluded	0.932	0.509‐1.707	0.820	0.681	0.344‐1.346	0.269
Desert	2.280	1.107‐4.696	0.025*	2.146	0.941‐4.893	0.070

Abbreviation: AJCC, Americant Joint Committee on Cancer.

## 4 DISCUSSION

In the present study, we show for the first time that the immune phenotype of OTSCC predicts early relapse and poor prognosis. Specifically, univariate and multivariate survival analysis showed that OTSCC patients with an immune‐desert phenotype had lower likelihood of survival compared to the other groups. Our findings were based on the results of a single, large cohort of 211 OTSCC patients treated by means of primary surgery with or without adjuvant therapies.

Solid tumors, including OTSCC, consist of a complex cellular ecosystem with a spatial organization, where a continuous interplay between cancer cells and tumor microenvironment takes place.^36^ Within the latter, research conducted in recent years has convincingly demonstrated that tumor immune microenvironment, in particular the TILs, play a critical role in cancer progression.^37^, ^38^ As the name suggests, TILs consist of lymphocytes that have invaded tumor tissues and are implicated in killing tumor cells. Despite the numerous studies that have been conducted to investigate the prognostic and predictive role of several immune‐related markers in OSCC, none of these has proven to be useful for adequate patient stratification.^21^, ^39^, ^40^, ^41^ With regard to TILs, accumulating evidence suggests that the assessment of these cells in histopathological specimens of solid tumors is a reliable and reproducible method, both by H&E stain and immunohistochemistry.^34^, ^42^, ^43^ Nevertheless, extensive methodological research is still needed to validate TILs markers for routine clinical use.

Regarding the prognostic role of TILs in OSCC, several immunohistochemical studies have revealed that increased levels of TILs are associated with favorable prognosis. In particular, the presence of high levels of CD^3+^ TILs at invasive tumor margin was associated with increased survival in OSCC patients.^21^ Low density of stromal CD^4+^ FOXP^3+^ TILs was identified as an independent prognostic marker for poor outcomes.^44^ Also, the presence of high levels of CD^8+^ TILs correlate with longer OS.^38^ Among other TILs subpopulations, a high Th17/Treg ratio was found to be associated with better outcomes in OSCC.^45^ Taken together, these results suggest that high densities of CD^3+^ (pan T cell marker), CD^4+^ (T helper cell marker), and CD^8+^ (T cytotoxic cell marker) TILs are independent factors for favorable prognosis. However, the mere quantification of TILs seems not to be an effective prognostic marker in most cancers including OSCC. Indeed, other studies have failed to show prognostic significance of specific TIL subpopulations in OSCC, limiting the reliability of the results reported in literature.^46^

A growing interest has emerged in the recent years regarding the potential importance of the spatial organization of TIL infiltrate in relation to cancer cells.^25^ The new paradigm for the classification of solid tumors, based on the distribution of immune cells, has recently emerged with the aim of improving the prognostic accuracy of TIL infiltrates.^27^ Hence, the present study aimed to investigate the prognostic role of immune phenotypes in OTSCC, staged according to both 7th and 8th editions of the AJCC Cancer Staging Manual.

In our study, a three‐type model for the immune phenotype^27^ was applied to split up the tumor specimens of our cohort, based on the distribution of immune cells. Some authors have highlighted how the number of immune phenotypes can vary from two to four and more, based on criteria used for the classification of tumor topography.^25^, ^28^, ^47^ Nevertheless, regardless of the classification system used, the immune‐desert phenotype is uniquely defined as the absence of immune cells in both the tumor parenchyma and the tumor stroma; therefore, the prognostic role of immune‐desert phenotype described in the present study is not influenced by the classification system being used. Furthermore, this subgroup shows distinctive molecular features that were identified by a molecular clustering analysis on a wider cohort of squamous cell carcinoma,^47^ thus setting this immune‐desert phenotype aside of other immune phenotypes. The current paradigm in cancer immunology predicts that the adaptive immune system represents an important defense mechanism against cancer.^27^ In this context, the paucity or lack of tumor T cell infiltration could be due to several reasons, such as the defective recruitment of antigens‐presenting cells, the lack of T cell activation or migration in tumor tissues, or altered cytokines' production.^48^ Regardless of the cause, several lines of evidence seem to suggest that solid tumor showing an immune‐desert phenotype (also called “cold tumors”) have a poor prognosis.^26^ Our results confirmed this hypothesis, suggesting that OTSCC patients showing immune‐desert phenotype lack of effective antitumor immune response, which is important for limiting the tumor growth and reducing the risk of recurrences.

Several authors have pointed out that the distinction between the immune‐inflamed and the immune‐excluded phenotypes is not clear‐cut, as a continuum of values related to the degree of immune cell infiltrate can be observed in the same specimen.^27^, ^49^ Our results confirm this observation, as the T cell density inside and outside the tumor hinders in many cases a clear classification of the specimen and the prognostic stratification of patients. This observation is due to the temporal ordering of the immune infiltration into the tumor tissue, due to the continuous evolution of the crosstalk between cancer and immune cells.^37^ Indeed, there is continuous and reciprocal communication between the tumor itself and microenvironment through cytokines, chemokines and cell‐cell interactions and the histologically based tumor‐immune phenotypes represent only a static description of this complex phenomenon.^27^

Another relevant aspect of our study is that the immune‐desert phenotype is the least common profile of OTSCC, representing 11.8% of the total. This is consistent with the data reported in solid tumors,^28^, ^36^ in which the immune‐desert profile is also associated with a significantly worse prognosis.^37^, ^47^ Furthermore, results from the survival analysis were consistent with the orientation of the recent literature. When applying the 7th edition of AJCC staging system to classify the OTSCC samples, we found that the immune‐desert phenotype was significantly correlated with a worse prognosis. In particular, results from multivariate analysis revealed a worse DSS (HR = 2.673; [CI: 95% 1.497‐4.773]; *P* = .001) and OS (HR = 2.591; [CI: 95% 1.468‐4.572]; *P* = .001). Since the 8th edition of AJCC staging system has been recently released, we decided to update our database accordingly and evaluated the parameters introduced by the new classification.^50^ Based on the data available in our database, a complete reclassification was possible for 139 samples. Survival analyses were then performed on this “restaged” subgroup, for which immune‐desert phenotype resulted again to be an independent prognostic factor for both DSS (HR = 2.280; [CI: 95% 1.107‐4.696]; *P* = .025) and OS (HR = 2.299; [CI: 95% 1.115‐4.742]; *P* = .024).

Based on the results obtained in this present work, a specific subgroup of patients with a different prognosis was identified. Additionally, our hypothesis was subjected to external validation using independent cohort of OTSCC patients from the TCGA database. The results were encouraging, confirming the rarity of the immune‐desert phenotype (5/76; 6.6%). Moreover, the limited number of cases prevented us from validating the study with a multivariate model, although the univariate analysis confirmed the prognostic implication of the immune‐desert phenotype. Our results regarding the prognostic role of TILs are in agreement with those recently reported by Heikkinen et al in a multicenter cohort of OTSCC patients.^51^ In particular, using a cut‐off point for TIL infiltration of 20%, it was found a subgroup of OTSCC patients (16.6%) characterized by a poor prognosis. Interestingly, the authors found a moderate interobserver agreement for the detection of tumors with low TILs (Cohen *κ* = 0.64). This data, although acceptable, might not be optimal and highlights the presence of a certain degree of uncertainty and variability in this method. In contrast, by applying the three‐type model for the immune phenotype, we found a smaller subgroup of OTSCC (11.8%), characterized by the absence of TILs in both the tumor parenchyma and stroma, that is, the immune‐desert phenotype. Using this definition, we obtained an almost perfect interobserver agreement (Cohen *κ* = 0.886),^52^ suggesting that the use of the immune phenotype model seems to be a reliable and accurate method to feature the role of TILs in tumor progression.^51^

There are wide clinical and translational implications of our results. The implementation of the use of the immune‐desert phenotype as a prognostic factor in the daily practice of oral pathology services is likely to be facilitated by the practical advantages of this technique, such as the use of standard H&E staining, the low inter‐observer variation, and the little extra time required. Furthermore, our results will inform further investigations of the molecular milieu responsible for the suppression of T‐cell immunity. Interestingly, recent studies have highlighted the contrasting role of the immune cells within the tumor microenvironment in OSCC.^21^, ^38^, ^40^, ^41^ Therefore, the study of the tumor‐immune phenotype in OSCC will require a better understanding of the molecular network governing the immunological response both in the tumor stroma and in the tumor nests.^53^

An important issue that must be considered when evaluating immune‐phenotype is the presence of extensive ulcerations and necrotic areas. Indeed, it is well known that a certain number of OSCCs present with ulcerated areas and, consequently, secondary inflammation.^54^ As previously stated, the areas of immune cell infiltrate associated with necrosis must be left out of the microscopic field or ignored for scoring the immune‐phenotype. Nevertheless, this aspect could make the evaluation of immune‐phenotype difficult.

The main limitations of the present study are the relatively low sample size of OTSCC patients with immune‐desert phenotype and its retrospective nature. Beside this, the results obtained provide important insights into the prognostic significance of the tumor‐immune profiles. In conclusion, evaluation of the immune‐desert phenotype is simple, inexpensive and can be used in daily practice with the aim of improving risk stratification of OTSCC patients, however further OTSCC cohorts should be evaluated to confirm such promising findings.

## CONFLICT OF INTEREST

The authors declare that they have no conflict of interests.

## AUTHOR CONTRIBUTIONS

Giuseppe Troiano: conceptualization, data curation, software, investigation, visualization, writing––original draft; Corrado Rubini: formal analysis, methodology, visualization, and validation; Lucrezia Togni: investigation, data curation, formal analysis, and project administration; Vito Carlo Alberto Caponio: data curation, software, and writing––original draft; Khrystyna Zhurakivksa: formal analysis, project administration, resources, and writing––original draft; Andrea Santarelli: funding acquisition, resources, project administration, and supervision; Nicola Cirillo: validation, writing––review and editing, supervision; Lorenzo Lo Muzio: funding acquisition, formal analysis, project administration, writing––review and editing, supervision; Marco Mascitti: conceptualization, investigation, formal analysis, writing––review, and editing.

## Supporting information

Supplementary Material

Supplementary Material

## Data Availability

The data that support the findings of this study are available from the corresponding author upon request.
