# Supplementary material for: The immune phenotype of tongue squamous cell carcinoma predicts early relapse and poor prognosis
Source: Cancer Med. 2020 Oct 13;9(22):8333–44. doi: 10.1002/cam4.3440 (PMC7666743; doi:10.1002/cam4.3440)
Supplement: Supplementary file 1 — Supplementary Material [file CAM4-9-8333-s001.docx]

**Supplemental Material 1: Results from Chi-square test among Immune-phenotype and the other clinic-pathological variables.**

| **Variable** | **Immune-phenotype** | | | |  | **Pearson’s chi-squared** | | |
| --- | --- | --- | --- | --- | --- | --- | --- | --- |
|  | **Inflamed** | **Excluded** | **Desert** | **Total** |  | **Value** | **df** | **Sig. two tails** |
| **Gender** |  |  |  |  |  |  |  |  |
| Male | 48 | 70 | 19 | 137 |  | 1.597 | 2 | 0.450 |
| Female | 29 | 39 | 6 | 74 |  |  |  |  |
| *Total* | 77 | 109 | 25 | 211 |  |  |  |  |
| **Grading** |  |  |  |  |  |  |  |  |
| G1 | 17 | 17 | 4 | 38 |  | 4.024 | 4 | 0.403 |
| G2 | 43 | 59 | 11 | 113 |  |  |  |  |
| G3 | 17 | 33 | 10 | 60 |  |  |  |  |
| *Total* | 77 | 109 | 25 | 211 |  |  |  |  |
| **7th AJCC edition** |  |  |  |  |  |  |  |  |
| Stage I | 17 | 28 | 6 | 51 |  | 1.886 | 6 | 0.930 |
| Stage II | 23 | 30 | 7 | 60 |  |  |  |  |
| Stage III | 15 | 26 | 7 | 48 |  |  |  |  |
| Stage IV | 22 | 25 | 5 | 52 |  |  |  |  |
| *Total* | 77 | 109 | 25 | 211 |  |  |  |  |
| **Perineural invasion** |  |  |  |  |  |  |  |  |
| No | 25 | 42 | 11 | 78 |  | 1.832 | 2 | 0.400 |
| Yes | 51 | 58 | 14 | 123 |  |  |  |  |
| *Total* | 76 | 100 | 25 | 201 |  |  |  |  |
