# Supplementary material for: The immune phenotype of tongue squamous cell carcinoma predicts early relapse and poor prognosis
Source: Cancer Med. 2020 Oct 13;9(22):8333–44. doi: 10.1002/cam4.3440 (PMC7666743; doi:10.1002/cam4.3440)

**Supplemental Material 2: Univariate and Multivariate overall survival for the immune-phenotype in the TCGA database. Gender, Staging and perineural invasion were used in the multivariate model. Kaplan-Meier curves for the univariate overall survival in TCGA database.**

| **Overall Survival** | | | | | | |
| --- | --- | --- | --- | --- | --- | --- |
|  | **Univariate** | | | **Multivariate** | | |
| **Variable** | **HR** | **95% CI** | **P-value** | **HR** | **95% CI** | **P-value** |
| Age | 1.004 | 0.977-1.032 | 0.790 |  |  |  |
| Gender |  |  |  |  |  |  |
| Female | 1 |  |  | 1 |  |  |
| Male | 1.596 | 0.813-3.130 | 0.174 | 0.564 | 0.240-1.326 | 0.189 |
| Grade |  |  |  |  |  |  |
| G1 | 1 |  | 0.230 |  |  |  |
| G2 | 1.808 | 0.532-6.143 | 0.343 |  |  |  |
| G3 | 2.824 | 0.786-10.144 | 0.111 |  |  |  |
| Staging system |  |  |  |  |  |  |
| Stage I | 1 |  | 0.002 | 1 |  | 0.156 |
| Stage II | 0.389 | 0.114-1.325 | 0.131 | 0.327 | 0.059-1.806 | 0.200 |
| Stage III | 0.246 | 0.065-0.931 | 0.039 | 0.330 | 0.068-1.604 | 0.169 |
| Stage IV | 1.333 | 0.449-3.956 | 0.604 | 1.039 | 0.269-4.008 | 0.956 |
| Perineural Invasion |  |  |  |  |  |  |
| No | 1 |  |  | 1 |  |  |
| Yes | 1.752 | 0.722-4.253 | 0.215 | 1.645 | 0.622-4.349 | 0.316 |
| Immune-phenotype |  |  |  |  |  |  |
| Inflamed | 1 |  | 0.003 | 1 |  | 0.177 |
| Excluded | 0.555 | 0.269-1.147 | 0.112 | 0.685 | 0.267-1.763 | 0.433 |
| Desert | 3.354 | 1.187-9.479 | 0.022 | 2.634 | 0.615-11.272 | 0.192 |


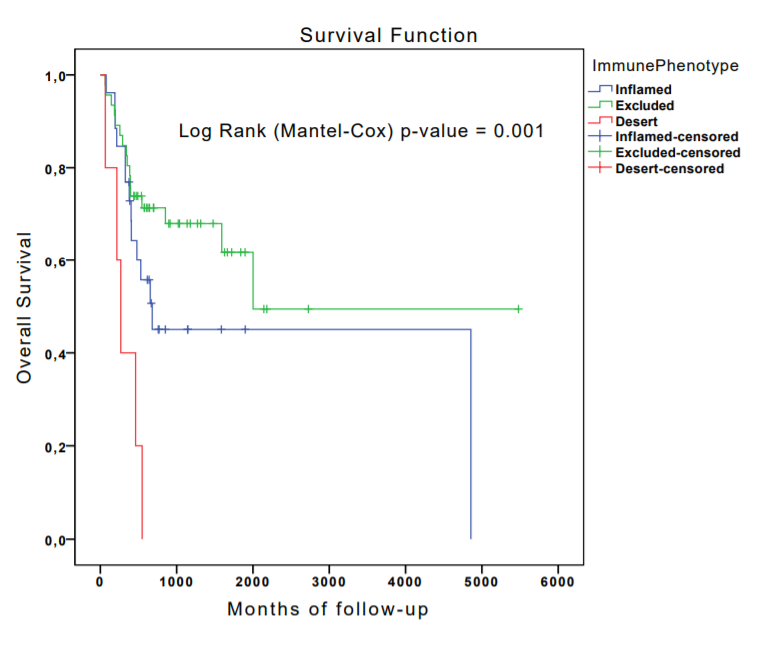

Supplement: Supplementary file 2 — Supplementary Material [file CAM4-9-8333-s002.docx]
